# Multifaceted interventions for improving spontaneous reporting of adverse drug reactions in a general hospital in China

**DOI:** 10.1186/s40360-017-0159-0

**Published:** 2017-06-26

**Authors:** Huan Fang, Xiaowen Lin, Jun Zhang, Zhen Hong, Kenji Sugiyama, Takao Nozaki, Tetsuro Sameshima, Susumu Kobayashi, Hiroki Namba, Tetsuya Asakawa

**Affiliations:** 1 Department of Pharmacy, Jinshan Hospital of Fudan University, No. 1508 Longhang Road, Shanghai, 201508 People’s Republic of China; 2 Department of Pharmacy, Jinshan Central Hospital, No 147 Jiankang Road, Shanghai, 201500 People’s Republic of China; 3 Department of Neurology, Huashan Hospital of Fudan University, No. 12 Urumchizhong Road, Shanghai, 200040 People’s Republic of China; 4 Department of Neurosurgery, Hamamatsu University School of Medicine, Handayama, 1-20-1, Higashi-ku, Hamamatsu-city, Shizuoka 431-3192 Japan

**Keywords:** Adverse drug reactions (ADRs), Spontaneous reporting (SR) system, Pharmacovigilance, Use and abuse of antibiotics

## Abstract

**Background:**

The present study investigates changes in spontaneous reporting (SR) compliance and ADR patterns following adoption of a new hospital SR system, and multiple interventions designed for its improvement use under modified drug administration guidelines.

**Methods:**

In total, 1389 ADR cases were reviewed. Cases were divided into two groups, cases from period 1 (*n* = 557, from January 2006 to June 2011) under the old SR system and cases in period 2 (*n* = 832, from July 2011 to December 2016) under the new SR system with multiple interventions to improve physician SR compliance. General information, drug information, and clinical manifestations were investigated and compared between periods.

**Results:**

Interventions for improved clinician training, education on knowledge, attitudes, and practices (KAP), and economic incentives substantially improved SR adherence. We also found that changing drug usage patterns (based on the new drug administration guidelines) greatly influenced ADR occurrence and type.

**Conclusions:**

We found the SR compliance can be improved by multifaceted interventions. Drug usage patterns also influence ADR occurrence, so programs tailored for rational use are essential. These results could lead to further improvements in the SR system for ADRs in China, and provide guidance for establishing better methods of pharmacovigilance.

## Background

Adverse drug reactions (ADRs) are defined as any harmful and unintended responses to an approved medication administered by the recommended route and at appropriate doses during clinical practice [1, 2]. ADRs are a major cause of morbidity and mortality and a contributor to rising healthcare costs [3–5]. ADRs are the 4th–6th leading cause of death in the United States [6]. Although ADRs are sporadic in individuals, they are inevitable in populations. It has been estimated that approximately 3.7–6.0% of patients experience ADRs during hospitalization [7, 8]. Such events are unavoidable because it is impossible to produce a medicine with no side effects. The ADR risk is influenced by patient sex and age, types of drugs prescribed, severity of the underlying disease, and number of drugs administered [9–13]. Pharmacovigilance aimed at identifying and quantifying the risks of drug administration is gradually improving, leading to a better understanding, estimation, and mitigation of ADRs [4, 14–16].

The spontaneous reporting (SR) system is the most common method of pharmacovigilance [17] and post-marketing signal detection [18, 19] and is a crucial contributor to drug safety throughout the world [20]. However, some SR systems are more effective than others. A highly effective system of pharmacovigilance can reduce under-reporting, and provides more useful information on ADR risks and methods for amelioration. The most important factors influencing SR efficacy are the knowledge, attitude, and practices (KAP) of physicians [19]. Previous studies have reported that insufficient knowledge regarding specific ADRs, poor appreciation of the importance of the SR system, and limited time to perform effective reporting may be obstacles to effective SR [21], which caused a high under-reporting, about 15.1% for the hospitalised patients [6]. Therefore, multifaceted interventions directed towards clinicians are very important. Several previous studies have reported that effective interventions such as financial incentives, training courses, improving reporting system and using electronic submission may significantly improve SR compliance by hospital physicians, and decrease the under-reporting [8, 19, 21].

In China, the first trial regulations for ADR reporting were established in 1999. These regulations were improved in 2004 with the release of the first version of Provisions for Adverse Drug Reaction Monitoring and Reporting. The guidance and supervisory recommendations of this version proved insufficient for drug safety control, so a new version was issued (July 2011), along with Special Measures for Controlling Antibiotics Use (April 2011). The newer version SR reporting system contains three substantial improvements over the first version: (1) all data must be reported and sent via internet, and traditional reporting methods (e.g., by letter, fax, or telephone in non-emergency cases) are not acceptable; (2) the database including documents and files should be administrated by computer; (3) the ADR surveillance center should have more authority to manage the SR system [22] []. And the main objective of the newer measures for controlling antibiotics use is to regulate the usage of antibiotics and to avoid abuse of antibiotics. In addition to adopt these new rules, our hospital also conducted several interventions designed to improve SR compliance among clinicians and promote a more rational use of drugs. The present study compares the SR data collected under the old regulations (without intervention) and under the new regulations (with intervention) to demonstrate the efficacy of specific interventions on SR compliance and clinical utility. Information included in the SR system regarding specific ADRs, such as type of drug prescribed, dosage form, and clinical manifestations, were investigated in both periods to assess if compliance and pharmacovigilance were improved in our hospital.

## Methods

### Setting and data source

All ADR cases in this study were collected during the period from January 2006 to December 2016 from the SR system of Jinshan hospital, a 700-bed clinical center with more than 943 doctors, nurses, and pharmacists located in the Jinshan district of southern Shanghai. Jinshan hospital is the only public and teaching medical center in Jinshan district. The reported ADR cases were first investigated by the hospital ADR surveillance unit in the pharmacy department, and then rechecked by the ADR surveillance center affiliated in this hospital. Cases with any uncertainty or mistakes found by the ADR surveillance center that could not be verified were sent back to the pharmacy unit for reinvestigation. Only verified cases (cases without any reporting mistakes and other suspicious reporting problems verified by the ADR surveillance center) were included in the present study.

All data were collected using the modified methods described in a previous study [22]. In brief, three types of data were collected: (1) general demographic information (patient sex, age, suspected medications, etc.), (2) temporal factors related to the ADR (time of occurrence, time of adoption of intervention measures, etc), (3) ADR-specific clinical information (symptoms, signs, laboratory results during occurrence, courses of these symptoms, treatments, outcomes, and the professional details of the reporting staff).

To calculate the reporting rates, including total ADR and serious ADR reporting rates, the total number of patients (including both outpatients and inpatients) during the investigating period was obtained from the hospital information system (HIS).

### Assessment criteria

The WHO-Uppsala Monitoring Center criteria [1] were employed to classify the level of causality as certain, probable/likely, possible, unlikely, conditional/unclassified, or inaccessible/unclassifiable. Serious ADRs were also identified according to the WHO-Uppsala Monitoring Center criteria, which includes life-threatening events, death, permanent disabilities, significant impairment of organ function, congenital abnormalities, prolonged hospitalization, hospital admission, and other important medical events that could cause the above outcomes if left untreated. General ADRs were defined as all the other ADRs that could not be classified as serious. Total ADRs included general and serious ADRs. Illnesses associated with ADRs were classified according to the WHO Adverse Reaction Terminology (WHO-ART) [22].

### Interventions

The ADRs and reporting rules were changed under the new guidelines. Measures to control antibiotic usage may impact on ADRs by improving prescribing, rather than an increase in SR of ADRs and how it was thought that this would impact on ADRs. These new regulations were complemented by interventions to improve SR compliance by clinicians. The related clinicians, clinical pharmacists, as well as the related nurses were involved in the interventions and implemented by an experienced clinical pharmacist. The interventions included (1) financial incentives, (2) training courses, (3) improvement of the computer system, (4) regular publishing of ADR information, (5) alerts on serious ADRs, and (6) regulation of antibiotic use. Efficient ADR reporting now results in a financial reward (20 RMB/time, about 3 USD/time) to the clinician, and the department with the best SR compliance is rewarded 8000 RMB (about 1200 USD) at the end of the year. Training courses were established to improve KAP regarding ADR reporting, the SR system, and reasonable use of medications. The computer system was improved to make the SR more convenient, and only the electronic format is now acceptable. Information is published quarterly to improve awareness of ADRs, and to update the detailed reporting achievements of each department. Enforcement measures for the reasonable use of antibiotics include limiting the number of antibiotics available to < 50, and having all department heads sign a document supporting antibiotic control. Further, all clinicians are now well-trained on guidelines and rules before being granted the right to prescribe and dispense antibiotics. The clinic pharmacists also evaluate antibiotic prescriptions periodically. All the interventions were implemented from the beginning of period 2 (see below).

### Classification of study periods

The cases included in this study were divided into two groups, period 1 and period 2. Cases reported under the old SR system (the old Provisions for Adverse Drug Reaction Monitoring and Reporting from January 2006 to June 2011) with no interventions were included in period 1. Cases reported from July 2011 to December 2016 under the new SR system and regulations with interventions were included in period 2. The total duration of both periods was 66 months.

### Statistics

All statistical analyses were conducted using SAS 9.4 software (SAS Institute, Cary, NC, USA). The level of significance (*p*) was set as 0.05 (two-tailed) for all statistical tests. Normality was tested by the Shapiro-Wilk and Kolmogorov-Smirnov tests. Since neither total ADRs nor serious ADRs were normally distributed, the differences in reporting rates (number of reported ADRs/total number of patients) and reported numbers between periods were therefore assessed by the non-parametric Wilcoxon rank-sum test.

## Results

### General information on ADRs during the two periods

There were 557 ADRs during period 1 (from January 2006 to June 2011), 21 of which were serious; and 832 ADRs during period 2 (July 2011 to November 2015), 258 of which were classified as serious (Table 1).

**Table 1:** General profile of ADRs in two periods

Gender/Age	Period 1		Period 2	
	Total ADRs ADRs	Serious ADRs	Total ADRs ADRs	Serious ADRs
Female	296	13	455	130
Male	261	8	377	128
Over 45	268	13	613	236
Below 45	289	8	214	17
Age unknown			5	5

There was no significant difference in the total reporting rate (*p* = 0.8023) between periods despite the total reporting number in period 2 was significantly higher than that of period 1 (*p* = 0.0086), and both the number of reported serious ADRs and the serious ADR reporting rate were significantly higher in period 2 than period 1 (both *p* < 0.0001). This enhanced reporting may have stemmed from the interventions instituted in period 2, which placed more emphasis on serious ADR reporting (Table 2).

**Table 2:** The number and rate of ADRs reporting in two periods

	Period1	Period2	*p* value
Total ADRs reporting number	557	832	0.0086
Monthly average of total ADRs reporting number	8.44	12.61	
Serious ADRs reporting number	21	258	<0.0001
Monthly average of serious ADRs reporting number	0.32	3.91	
Total ADRs reporting rate	0.0128%	0.011426%	0.8023
Serious ADRs reporting rate	0.0005%	0.003543%	<0.0001

### Dosage forms and drugs related to ADRs

The most common dosage forms and drugs associated with ADRs in the SR database were investigated and compared between periods. In period 1, “Injection preparations” were most likely to cause ADRs of any severity (*n* = 377, 59.0% of total ADRs) and serious ADRs (*n* = 13, 54.2%). In period 2, “Oral dosage forms” were most likely to cause ADRs of any severity (*n* = 646, 61.35% of total ADRs) and serious ADRs (*n* = 264, 76.97%) (Table 3).

**Table 3:** The frequency of dosage forms causing ADRs

Dosage forms	Period 1		Period 2	
	Total ADRs	Serious ADRs	Total ADRs	Serious ADRs
Injection preparations	377 (59.0%)	13 (54.2%)	364 (34.6%)	68 (19.8%)
Oral dosage forms	228 (35.7%)	11 (45.8%)	646 (61.4%)	264 (77.0%)
Other dosage forms	34 (5.3%)	0	43 (4.1%)	11 (3.2%)

The top three classes of drugs most frequently associated with ADRs in period 1 were antibiotics (*n* = 339, 53.1%), Chinese patent medicines (*n* = 91, 14.2%), and cardiovascular drugs (*n* = 34, 5.3%). In period 2, the drugs most frequently implicated in ADRs were antibiotics (*n* = 228, 21.5%), Chinese patent medicines (*n* = 171, 16.1%) and non-steroidal anti-inflammatory drugs (NSAIDs, *n* = 154, 14.5%), (Table 4). Considering only serious ADRs, the top three drugs in period 1 were antibiotics (*n* = 5, 20.8%), antilipemics (*n* = 3, 12.5%), and plasma expanders (*n* = 3, 12.5%); in period 2, the drugs most commonly associated with severe ADRs were NSAIDs (*n* = 107, 31.7%), antibiotics (*n* = 35, 10.4%), and cardiovascular drugs (*n* = 34, 10.1%) (Table 5). The drop of antibiotics in serious ADRs in period 2 likely resulted from increased emphasis on controlled usage.

**Table 4:** The frequency of culprit drug of total ADRs in two periods

Period 1		Period 2	
Drug	No.	Drug	No.
Antibiotics	339 (53.1%)	Antibiotics	228 (21.5%)
Cephalosporin	101	Cephalosporin	83
Quinolone	89	Quinolone	65
Macrolide	42	Macrolide	29
Penicillin	38	Penicillin	21
Lincosamide	23	Nitroimidazole	7
Nitroimidazole	17	Lincosamide	6
Aminoglycosides	7	Glycopeptide	9
Glycopeptide	7	Fosfomycin	1
Fosfomycin	6	Antifungal	1
Antifungal	4	Tetracycline	1
Antituberculotic	4	Antituberculotic	2
Sulfa	1	Other	3
Chinese patent medicine	91 (14.2%)	Chinese patent medicine	171 (16.1%)
Cardiovascular	34 (5.3%)	NSAIDs	154 (14.5%)
CCBs	5	Conventional	139
ACEI/ARB	5	Cox-2 inhibitor	15
Antilipemic	23	Cardiovascular	89 (8.4%)
Antiarrhythmic drugs	1	Antihypertensive	37
NSAIDs	31 (4.9%)	Antilipemic	13
Conventional	25	Antiarrhythmic drugs	14
Cox-2 inhibitor	6	Nitrates	16
CNS drugs	17 (2.7%)	Cardiotonic	9
Antidepressant	7	Antithrombotic	43 (4.1%)
Improving CNS metabolism	4	CNS drugs	51 (4.8%)
CNS muscle relaxant	2	Antidepressant	21
Anticonvulsant	4	Improving CNS metabolism	2
Antiviral	16 (2.5%)	CNS muscle relaxant	4
Gastrointestinal	15 (2.4%)	Anticonvulsant	3
PPI	6	Other CNS drug	21
Others	9	Endocrine	37 (3.5%)
Immunoregulation	11 (1.7%)	Gastrointestinal	34 (3.2%)
BAs	11 (1.7%)	PPI	20
Cytotoxicity	7 (1.1%)	Others	14
Respiratory	4 (0.6%)	Opioid	26 (2.5%)
Antithrombotic	4 (0.6%)	BAs	17 (1.6%)
Endocrine	3 (0.5%)	Antiviral	16 (1.5%)
Hormone	2 (0.3%)	Cytotoxicity	15 (1.4%)
Others	54 (8.5%)	Diuretic	15 (1.4%)
		Hormone	15 (1.4%)
		Antigout	11 (1.0%)
		Respiratory	6 (0.6%)
		Immunoregulation	5 (0.5%)
		Others	127 (12.0%)

**Table 5:** The frequency of culprit drug of serious ADRs in two periods

Period 1		Period 2	
Drug	No.	Drug	No.
Antibiotics	5 (20.8%)	NSAIDs	107 (31.7%)
Cephalosporin	2	Conventional	97
Lincosamide	1	Cox-2 inhibitor	10
Sulfa	1	Antibiotics	35 (10.4%)
Cytotoxicity	1	Cephalosporin	13
Antilipemics	3 (12.5%)	Quinolone	18
Plasma expander	3 (12.5%)	Lincosamide	4
BAs	2 (8.3%)	Cardiovascular	34 (10.1%)
CNS drugs	2 (8.3%)	Antihypertensive	22
Chinese patent medicine	1 (4.2%)	Antilipemic	5
ACEI/ARB	1 (4.2%)	Antiarrhythmic	4
Antithrombotic	1 (4.2%)	Nitrates	1
Endocrine	1 (4.2%)	Cardiotonic	2
Immunoregulation	1 (4.2%)	Chinese patent medicine	27 (8.0%)
Others	4 (16.7%)	Antithrombotic	22 (6.5%)
		CNS drugs	18 (5.3%)
		Endocrine	17 (5.0%)
		PPI	11 (3.3%)
		Hormone	8 (2.4%)
		Others	59 (17.5%)

### Clinical manifestations of ADRs

In period 1, the top three system and organ dysfunctions associated with total ADRs were skin and appendage disorders (*n* = 299, 44.4%), body as a whole-general disorders (*n* = 109, 16.2%), and gastro-intestinal system disorders (*n* = 103, 15.3%). In period 2, the rank order was gastro-intestinal system (*n* = 272, 27.9%), skin (*n* = 208, 21.3%), and body as a whole-general (*n* = 146, 15.0%) (Table 6). Considering only serious ADRs, the top three in period 1 were whole-general (*n* = 10, 47.6%), liver and biliary (*n* = 6, 28.6%), and skin (*n* = 2, 9.5%); and the top three in period 2 were gastro-intestinal (*n* = 129, 45.0%), liver and biliary (*n* = 81, 28.2%). and whole-general (*n* = 25, 8.7%) (Table 7). The increase in gastro-intestinal system disorders from serious ADRs in period 2 may be the result of more reports relating to NSAIDs.

**Table 6:** The involved organs frequency of total ADRs in two periods

Period 1		Period 2	
Involved systems and organs	No.	Involved systems and organs	No.
Skin and appendages disorders	299 (44.4%)	Gastro-intestinal system disorders	272 (27.9%)
Body as a whole-general disorders	109 (16.2%)	Skin and appendages disorders	208 (21.3%)
Gastro-intestinal system disorders	103 (15.3%)	Body as a whole-general disorders	146 (15.0%)
Central & peripheral nervous system disorders	47 (7.0%)	Liver and biliary system disorders	108 (11.1%)
Liver and biliary system disorders	24 (3.6%)	Central & peripheral nervous system disorders	73 (7.5%)
Vascular (extracardiac) disorders	24 (3.6%)	Heart rate and rhythm disorders	43 (4.4%)
Application site disorders	19 (2.8%)	Urinary system disorders	20 (2.1%)
Heart rate and rhythm disorders	18 (2.7%)	Vascular (extracardiac) disorders	17 (1.7%)
Others	31 (4.6%)	Metabolic and nutritional disorders	15 (1.5%)
		Application site disorders	11 (1.1%)
		Respiratory system disorders	10 (1.0%)
		Platelet, bleeding & clotting disorders	10 (1.0%)
		Others	43 (4.4%)

**Table 7:** The involved organs frequency of serious ADRs in two periods

Period 1		Period 2	
Involved systems and organs	No.	Involved systems and organs	No.
Body as a whole-general disorders	10 (47.6%)	Gastro-intestinal system disorders	129 (45.0%)
Liver and biliary system disorders	6 (28.6%)	Liver and biliary system disorders	81 (28.2%)
Skin and appendages disorders	2 (9.5%)	Body as a whole-general disorders	25 (8.7%)
Platelet, bleeding & clotting disorders	2 (9.5%)	Metabolic and nutritional disorders	12 (4.2%)
Resistance mechanism disorders	1 (4.8%)	Urinary system disorders	9 (3.1%)
		Heart rate and rhythm disorders	6 (2.1%)
		Central & peripheral nervous system disorders	5 (1.7%)
		Others	20 (7.0%)

## Discussion

The present study focused on the effects of interventions accompanying the introduction of a new SR system and the new Special Measures for Controlling Antibiotics Use on SR compliance. Our data revealed an obvious improvement in SR compliance after implementing multiple intervention measures. Pharmacovigilance is indispensable for safe drug administration; however, SR systems differ among regions and countries due to region-specific predominant disease distributions, culture, medical educational programs, economic status, and pharmaceutical company marketing. It is thus useful to collect and analyze information from different countries to help both government regulators and investigators establish appropriate rules to improve the efficacy and safety of drug administration. Similar studies have been conducted in Spain [19, 21] and Korea [8, 23], but to the best of our knowledge, there is no previous study regarding interventions for improving the SR system in China. The present study collected and analyzed SR system data spanning 10 years from a medical center in Shanghai, which is representative of many such institutions across the country. Thus, we believe that our conclusions are applicable to other regional hospitals in China.

Previous studies in other countries have demonstrated that regulations or interventions can improve pharmacovigilance and enhance ADR reporting [19, 21, 23–26]. We used the same experimental design as in these previous studies [19, 21] to compare the SR compliance and ADR patterns between two periods, before and following the implementation of interventions. We found no sex difference in either total or serious reported ADRs, in accordance with previous studies (Table 1, *p* > 0.05) [10, 20, 27–29]. More serious ADRs were found in patients older than 45, particularly in period 2. This result is also consistent with previous findings that history of ADRs or hospital admissions caused by ADRs increase with age [16, 30–32]. This association may be explained by the increase in the number of drugs taken with age [30]. In both periods, injection and oral were the dosage forms causing the most and serious ADRs reported (Table 3). Antibiotics were most frequently associated with ADRs, and skin and appendage disorders were the most and second frequent manifestations in period 1 and period 2 respectively (Table 4), again in accordance with previous results from other countries [8, 23, 33, 34].

Although there was no significant difference in the total reporting rate between periods (Table 2, *p* > 0.05), the total reporting number, along with the reported number and related reporting rate for serious ADRs were significantly enhanced during period 2 (Table 2, *p* < 0.05), suggesting that the interventions were indeed effective. These improvements were likely due to the new SR system that encourages and rewards reporting of serious ADRs (or the reporting of serious ADRs was neglected in period 1.). However, our study found that only the reporting of serious ADRs was enhanced, while a previous study found enhanced reporting of all (total) and serious ADRs [19]. This difference may be the result of the different interventions used, as our interventions were based on new guidelines (revised Provisions for Adverse Drug Reaction Monitoring and Reporting), while the previous study used the same guidelines for both periods. One explanation we did not get a higher total reporting rate in the present study is that we calculated the total reporting rate using total number of patients as the denominator, which remarkably increased during the period 2. We know there is 5.5-year difference between periods. Use of safer and more effective medicine, enforcement measures for the reasonable use of medicine, and progression in medical technology in period 2 may potentially reduce ADRs. In this regard, although the patients increased in period 2, the actual occurrence rate of ADR may reduce. Thus, to select an appropriate index to evaluate the reporting rate is a problem in the future analogous study. We consider that calculating the index using number of reported ADRs/total number of patients with ADRs is more reasonable for the reporting rate; however, the total number of patients with ADRs is difficult to obtain in the actual practice. Nonetheless, we achieved the same result; an intervention based on education and economic incentives improves SR compliance for ADRs. Data of the present study is very dependent on the areas. It is very interesting to compare the present data with the SR from the other areas of China. Although there is no report available to compare the data between two periods in the other areas, we found only two reports regarding the SR system in China available. Li et al investigated the SR system Shanghai Pediatric Population and got a reporting rate for serious ADRs were 2.16% [35], another study in Chinese reported that 5.11% (71 of 1390 cases) of serious ADRs were reported in Beijing between Jan, 2009 and Dec, 2012 [36] The data higher than our data in period 1 (3.77%), but lower than our data in period 2 (31.01%). We expect more analogous studies in the other areas of China can be found in the future.

Although antibiotics were the most frequent cause of ADRs in both periods, the fraction of antibiotics was markedly lower in period 2 compared to period 1 (21.5% vs. 53.1%). In contrast, more NSAIDs were associated with ADRs in period 2 compared to period 1 (14.5% vs. 4.9%) (Table 4). The pattern of serious ADRs also differed between periods. NSAIDs were the most frequent cause in period 2 (31.7%) while antibiotics were the most frequent cause in period 1 (20.8%) (Table 5). Accordingly, the most common administration route for ADRs also changed. Compared to period 1, oral dosages forms increased while injections decreased in period 2 (Table 3). These changes in the rank order of administration routes were due to changes in the drug administration pattern. In Chinese hospitals, most antibiotics for inpatients are administrated via injection, whereas NSAIDs are administered orally. Thus, changes in the predominant administration route associated with ADRs were the result of reduced usage of antibiotics during period 2. Moreover, clinical manifestations also changed. The most common manifestations were skin and appendage disorders in period1; however, the rate was reduced to 21.3% in period 2 from 44.4% in period 1. Conversely, gastro-intestinal system disorders rose to the most frequent manifestation in period 2 (27.9% vs. 15.3% in period 1) (Table 6). Changes in the patterns of serious ADRs were even more remarkable. The most common serious manifestations were gastro-intestinal system disorders (45.0% of serious ADRs in period 2), while the frequency of whole-general disorders dropped to third in period 2 from first in period 1 (8.7% vs. 47.6%) (Table 7). The changes in involved organs (Tables 6 and 7) were in accordance with the changes in the culprit drugs (Tables 4 and 5), as well as the administration route (Table 3). Again, these results can be explained by the reduction in antibiotic usage. We thus propose that the changes in ADR patterns resulted from improved measures for controlling antibiotic use and abuse, along with our interventions and the improved SR system. Consistent with previous studies, our results show that the frequency, severity, and patterns of ADRs were greatly affected by the drug usage pattern [8, 20].

## Conclusions

We compared SR system compliance and ADR patterns before and after interventions designed to promote better SR system use among clinicians in China. We found the SR compliance can be improved by factors like KAP, education, performance of the SR system (i.e., internet-based with regular analysis/dissemination of information regarding ADRs, etc.) and appropriate economic incentives. On the other hand, drug usage patterns also influence ADR occurrence, so programs tailored for rational use are essential. These results could lead to further improvements in the SR system for ADRs in China, and provide guidance for establishing better methods of pharmacovigilance.

## Abbreviations

ACEI: Angiotensin-converting-enzyme inhibitor
ADRs: Adverse drug reactions
ARB: Angiotensin receptor blocker
BAs: Biologic agents
CCBs: Calcium-channel blockers
CNS: Central nervous system
HIS: Hospital information system
KAP: Knowledge, attitude, and practices
NSAIDs: Non-steroidal anti-inflammatory drugs
PPI: Proton pump inhibitor
SR: Spontaneous reporting
WHO-ART: WHO Adverse Reaction Terminology
